# Takotsubo cardiomyopathy associated with pneumatosis cystoides intestinalis and postprandial hypoglycemia of anorexia nervosa

**DOI:** 10.1002/pcn5.33

**Published:** 2022-08-03

**Authors:** Shinichiro Ochi, Noboru Sano, Jun‐ichi Iga, Shu‐ichi Ueno

**Affiliations:** ^1^ Department of Neuropsychiatry Ehime University Graduate School of Medicine, Toon Ehime Japan; ^2^ Futaiwa Hospital Yawatahama Ehime Japan; ^3^ Present address: Department of Neuropsychiatry Ehime University Graduate School of Medicine, Shitsukawa Toon Ehime Japan

**Keywords:** anorexia nervosa, hypoglycemia, pneumatosis cystoides intestinalis, takotsubo cardiomyopathy

## Abstract

Hypoglycemia is not rare in anorexia nervosa (AN). Takotsubo cardiomyopathy (TCM) is characterized by extensive akinesis of the apical region with hypercontraction of the basal segment of the ventricle in the absence of coronary artery disease. Its mechanism is not fully understood, but hypoglycemia is considered one of the physical factors. Pneumatosis cystoides intestinalis (PCI) is a rare disease characterized by multiple gaseous cysts in the intestinal wall. PCI sometimes causes an absorption defect. The case of a 48‐year‐old woman with AN with PCI and TCM that developed after a postprandial hypoglycemic coma is reported. When the patient was admitted to our hospital, her abdominal X‐ray showed a confluent image of grapes, and computed tomography showed gaseous cysts in the intestinal wall from the ascending colon to the transverse colon. PCI was then diagnosed. About 7 days after admission, she developed hypoglycemic coma. However, she recovered from the coma and on the next day she became suddenly hypotensive, with the electrocardiogram showing T‐wave inversion. Echocardiography then showed akinesis around the apex and hypercontraction of the basal segments, and TCM was diagnosed. Severe AN with PCI may cause more severe hypoglycemia, resulting in TCM.

## Graphical abstract

A patient with anorexia nervosa with pneumatosis cystoides intestinalis developed takotsubo cardiomyopathy after a postprandial hypoglycemic coma. When chest pain and dyspnea with hypotension develop after hypoglycemia, clinicians should consider the possibility of takotsubo cardiomyopathy and perform electrocardiography and echocardiography quickly.

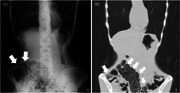

## BACKGROUND

Anorexia nervosa (AN) is a complex psychiatric illness that is often associated with general debility and medical disorders. It has been reported that patients with AN have many abnormal test results, such as hypoprealbuminemia, leukopenia, metabolic alkalosis, electrolyte imbalance, hypoglycemia, and osteoporosis.^1^ A previous study reported that the prevalence of hypoglycemia was greater than 40% in severe AN,^2^ therefore hypoglycemia is not rare in AN. Takotsubo cardiomyopathy (TCM) is characterized by transient apical wall motion abnormalities of the left ventricle, with compensatory hyperkinesis of the basal walls, producing ballooning of the apex with systole in the absence of coronary artery disease.^3^, ^4^ Its mechanism is not fully understood, but hypoglycemia is considered one of the physical factors.^5^, ^6^ Pneumatosis cystoides intestinalis (PCI) is a rare disease characterized by multiple gaseous cysts in the intestinal wall. PCI sometimes causes absorption defects such as diarrhea.^7^, ^8^ It has been reported that patients with AN can develop TCM^9^, ^10^, ^11^, ^12^ or PCI,^13^ but, to the best of our knowledge, a case of AN with both TCM and PCI has not been previously reported. The case of a 48‐year‐old woman with AN and PCI who developed TCM after a postprandial hypoglycemic coma is reported. Informed consent of this case presentation for publication was obtained from the patient.

## CASE PRESENTATION

**Figure 1:**
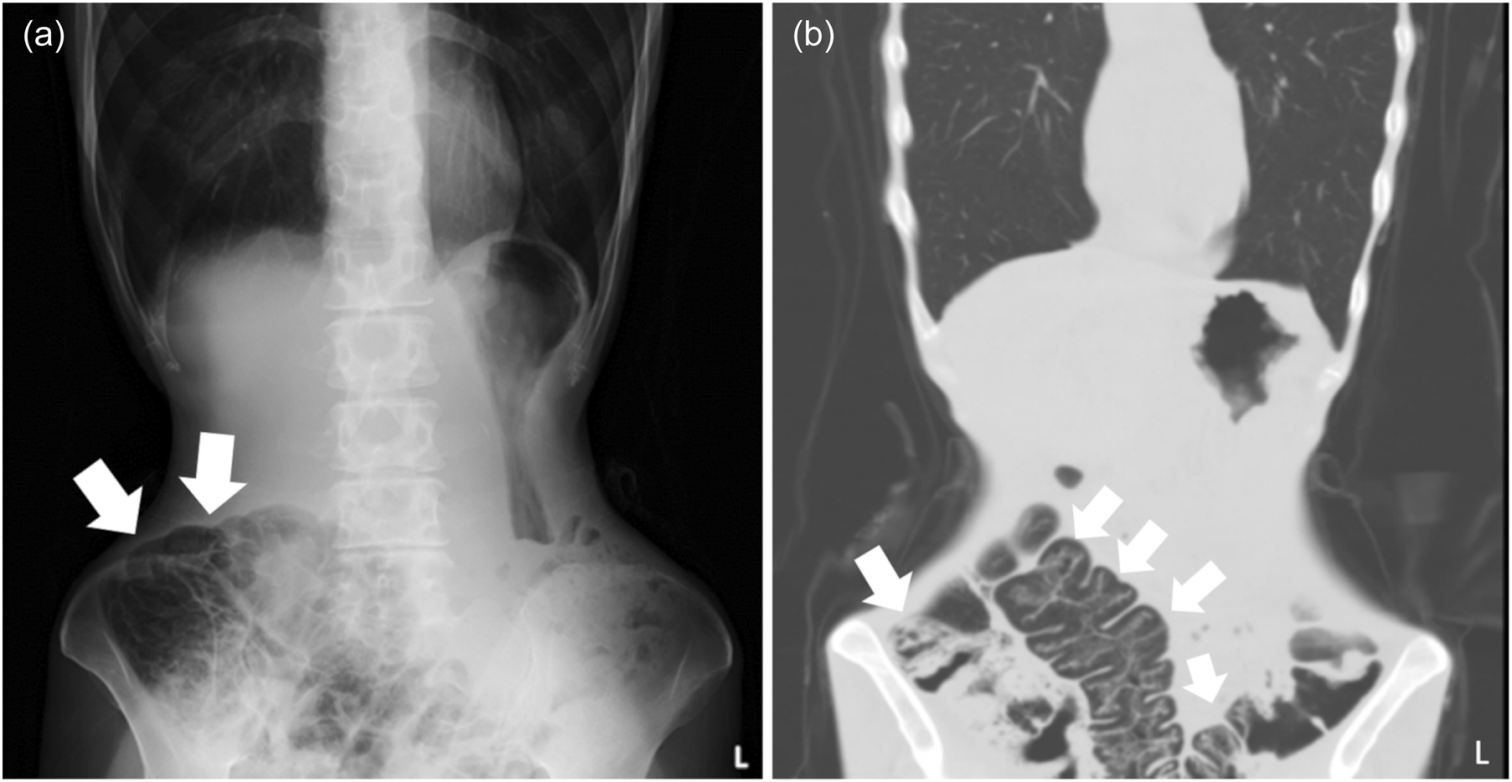
Abdominal X‐ray and abdominal CT of PCI. Abdominal X‐ray (a) shows a confluent image of grapes (arrows) and abdominal CT (b) shows cysts in the intestinal wall from the ascending colon to the transverse colon (arrows).

**Figure 2:**
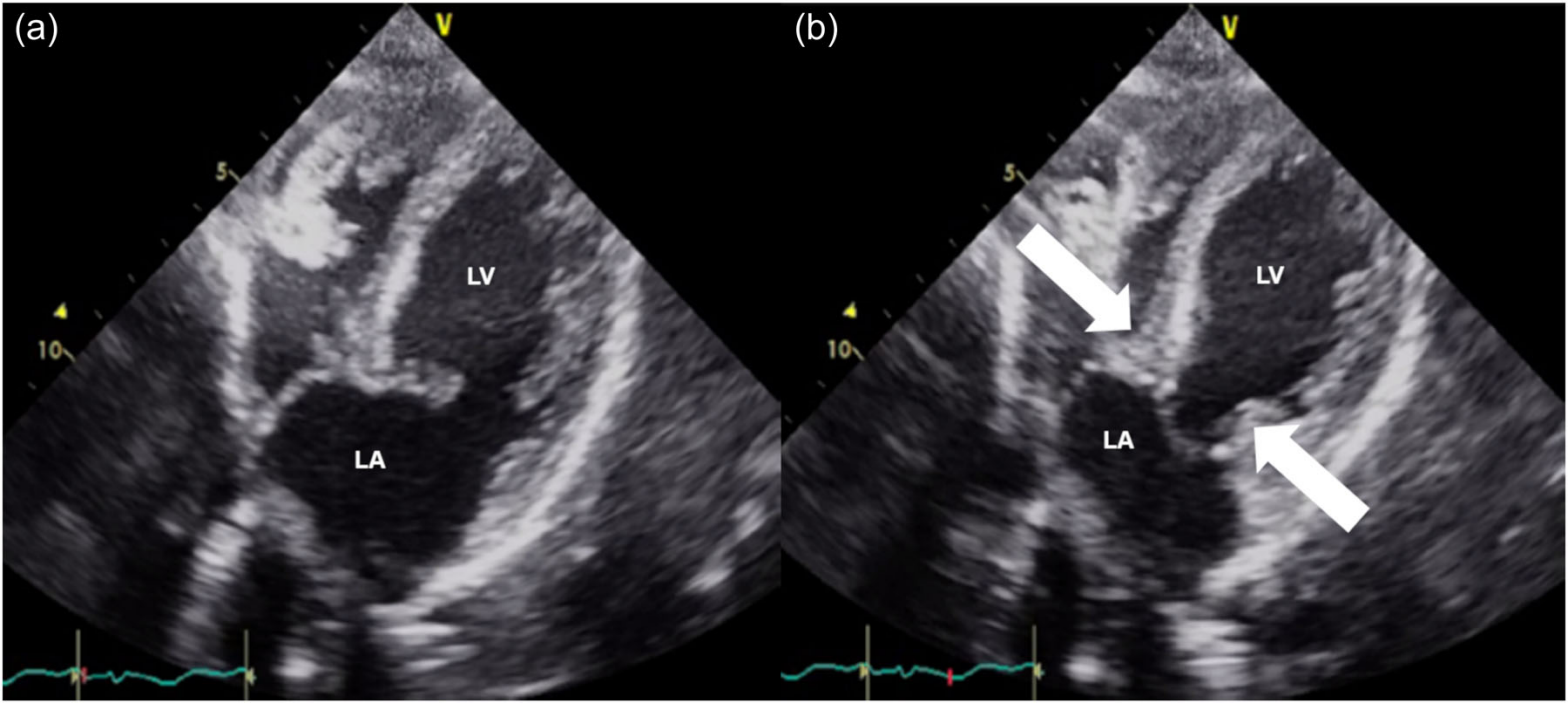
Echocardiography of TCM. Echocardiography shows akinesia around the apex and hypercontraction of the basal segments (arrows): (a) diastolic phase and (b) systolic phase. LA, left atrium; LV, left ventricle

A 48‐year‐old woman had a previous history of chronic obstinate constipation. When she was 42 years old, she had insomnia, and she then started binge eating and vomiting, and her weight gradually decreased. However, she was performing her job without problems. About 5 years after onset, she restricted her foods or only had noncaloric foods, and her body weight decreased markedly. Her parents therefore brought her to our hospital. At the first visit, she showed a distorted body image, denial of the seriousness of her low body weight, and difficulty walking, and her body mass index (BMI) was 12.2 kg/m^2^. Because of these findings, AN was diagnosed, and from her life history we excluded comorbid other psychiatric disorders such as borderline personality disorders. She was referred to our hospital for admission, but initially refused, although she was admitted to our hospital 2 weeks later. At the time of admission she showed marked emaciation and could not maintain the upright position. Her hospitalization was involuntary and we explained to her and her family that we estimated about 4 months for her hospital stay at admission. Her blood pressure was 119/78 mmHg and her BMI was 11.2 kg/m^2^. Blood tests showed hypoalbuminemia (3.7 g/dL, normal range 3.9–4.9 g/dL), hyponatremia (123 mmol/L, normal range 139–149 mmol/L), elevated hepatic enzymes (aspartate transaminase [AST] 153 U/L (normal range 9–37 U/L), alanine transaminase (ALT) 105 U/L (normal range 3–49 U/L), γ‐glutamyl transpeptidase (γ‐GTP) 70 U/L (normal range 6–71 U/L)), mild hypoglycemia (glucose 67 mg/dL, normal range 70–110 mg/dL), and hypothyroidism (thyroid stimulating hormone 5.830 μIU/mL (normal range 0.500–5.00 μIU/mL), free thyroxine 1.27 ng/dL (normal range 0.90–1.70 ng/dL), free triiodothyronine 1.21 pg/dL (normal range 2.3–4.0 pg/dL)), but inorganic phosphorus (IP) was within the normal range (3.0 mg/dL, normal range 2.2–5.3 mg/dL). Since her abdominal X‐ray showed a confluent image of grapes (Figure 1a), abdominal computed tomography (CT) was performed and showed cysts in the intestinal wall from the ascending colon to the transverse colon (Figure 1b). PCI was then diagnosed. After admission, the patient seemed emotionally stable. She was started on a diet of 500 kilocalories per day to prevent refeeding syndrome, and her intake was gradually increased. Three days after admission, her intake was increased to 700 kilocalories per day. Seven days after admission, her blood test results showed rapidly increasing hepatic enzymes (AST 991 U/L, ALT 717 U/L, γ‐GTP 162 U/L), hypokalemia (3.5 mmol/L, normal range 3.8–4.8 mmol/L), IP 2.8 mg/dL, and glucose 45 mg/dL. Developing refeeding syndrome was therefore suspected and her intake was decreased to 500 kilocalories per day. The following day, she suddenly developed postprandial hypoglycemia and hypoglycemic coma, and she was transferred to the intensive care unit. Her blood test results showed further increases in hepatic enzymes (AST 1858 U/L, ALT 955 U/L, γ‐GTP 160 U/L) and glucose fell to undetectable levels. She recovered from the coma on the same day. However, the next day, her blood pressure suddenly dropped to 64/34 mmHg, and the electrocardiogram showed markedly long QTs and T‐wave inversion in leads II, III, aVF, and V2‐V6. Echocardiography showed a large akinetic area around the apex and hypercontraction of the basal segments (Figure 2). TCM was then diagnosed. She was kept on bed rest and given intravenous fluids to prevent worsening heart failure. The PCI resolved within a few weeks, and her cardiac function gradually normalized within about 1 month. Her dietary intake was gradually increased to treat AN and her general status gradually improved. The pace for increasing caloric intake was slower than usual to avoid TCM relapsing, thus it took a long time to reach her goal weight. About 7 months after admission, she was discharged from the hospital. She did not react emotionally during her hospitalization, and we never used behavioral restrictions on her during the hospitalization.

## DISCUSSION AND CONCLUSION

The case of a 48‐year‐old woman with AN, PCI, and TCM that developed after a postprandial hypoglycemic coma was described. This is the first report of an AN patient with both PCI and TCM.

PCI is a rare disease that is not fully understood because it is a condition that may be associated with several diseases. The pathogenesis of PCI is unclear, but previous reports suggested that the causes of PCI were (1) mechanical factors, such as bowel ischemia, inflammatory bowel disease, surgery, and trauma, (2) pulmonary factors, such as chronic obstructive pulmonary disease, interstitial pneumonia, and asthma, (3) bacterial factors, such as clostridium infection, and (4) chemical or nutritional factors, such as chemotherapy, α‐glucosidase inhibitors, and malnutrition.^8^, ^14^ In this case, malnutrition from AN would cause PCI. Furthermore, in AN, it has been suggested that the pathogenesis of PCI may be mucosal disruption, which seems due to increased luminal pressure caused by self‐induced vomiting and obstinate constipation.^15^ In the present case, the patient had chronic obstinate constipation and began binge eating and self‐induced vomiting. Thus, one of the pathogenesis of PCI in her case may seem to have a similar mechanism to this previous report, therefore both malnutrition and mucosal disruption may have caused PCI in her case.

TCM is characterized by transient left ventricular apical wall motion abnormalities and chest pain with electrocardiographic changes, such as ST elevation and T‐wave inversion, without significant coronary artery disease. The pathogenesis of TCM is not fully understood, but a catecholamine surge seems to play a major role.^16^, ^17^ Recently, Yamamoto reported TCM induced by quite low dose epinephrine.^18^ It is known that the stress of hypoglycemia increases plasma catecholamine levels,^19^ and it has been reported that a patient with AN recovered from coma due to severe hypoglycemia the next day, developed hypotension, and was diagnosed with TCM.^10^

In the present case, the patient suddenly developed hypotension on the day after hypoglycemic coma and TCM was then diagnosed. This suggests that TCM developed with severe hypoglycemia as a trigger.

Regarding severe hypoglycemia with AN, a previous report suggested that postprandial reactive hypoglycemia could result from high insulin sensitivity.^20^ Furthermore, it has been reported that a patient with AN developed reactive hypoglycemia with characteristic insulin secretion, and rapid refeeding during the early treatment course could cause both excess insulin secretion from the very early phase and reactive postprandial hypoglycemia.^21^ Brown reported a case of Wernicke encephalopathy and TCM induced by refeeding syndrome in AN.^22^ They suggested that most complications of refeeding syndrome could be caused by electrolyte abnormalities such as hypophosphatemia, resulting in abnormal QT interval. Furthermore, refractory hypoglycemia including refeeding syndrome would be associated with cardiac complications such as TCM and heart failure.^23^, ^24^

Regarding the association between PCI and hypoglycemia, Asanuma reported a patient with hypoglycemia that was thought to be due to malabsorption and liver insufficiency, which might have been caused by PCI.^25^

In the present case, insulin secretion may have occurred due to refeeding, while storage of glycogen in the liver was minimal due to a long period of starvation and PCI may have caused malabsorption, in addition to the reduction of caloric intake. These complex factors may have caused severe postprandial hypoglycemia in the present case.

On the other hand, emotional stress can sometimes cause TCM, especially in cases comorbid with severe emotional dysregulation disorders such as borderline personality disorders.^26^, ^27^, ^28^ In this case, the patient did not identify any significant problems in her life history which we diagnosed borderline personality disorders, and she seemed emotionally stable during her hospitalization. However, the hospitalization was involuntary, and this may have caused her some emotional stress at the beginning of the hospitalization. This emotional factor might have affected the pathogenesis of TCM.

In conclusion, a case of AN with PCI and TCM was reported. In the self‐induced vomiting type of AN with comorbid chronic constipation, clinicians should consider gastrointestinal examinations, such as abdominal X‐rays and abdominal CT, and be particularly careful about hypoglycemia. Furthermore, when chest pain and dyspnea with hypotension develop after hypoglycemia, clinicians should consider the possibility of TCM and urgently perform electrocardiography and echocardiography.

## AUTHOR CONTRIBUTIONS

SO wrote original draft. NS was involved in data acquisition. JI and SU contributed to the editing and reviewing of the final manuscript. All authors approved the final manuscript and are responsible for the paper.

## CONFLICT OF INTEREST

The authors declare no conflict of interest.

## ETHICS APPROVAL STATEMENT

We obtained written consent from the patient to publish this case presentation.

## PATIENT CONSENT STATEMENT

We obtained written consent from the patient to publish this case presentation.

## CLINICAL TRIAL REGISTRATION

N/A.

## Data Availability

The data for this case report are available from the corresponding author on reasonable request.
